# Error Recovery Using Cooperative ARQ in Energy-Harvesting Wireless Sensor Networks with Data Allocation

**DOI:** 10.3390/s26082322

**Published:** 2026-04-09

**Authors:** Ikjune Yoon

**Affiliations:** Division of AI Computer Science and Engineering, Kyonggi University, 154-42 Gwanggyosan-ro, Yeongtong-gu, Suwon-si 16227, Republic of Korea; ijyoon@kyonggi.ac.kr

**Keywords:** wireless sensor networks, energy harvesting, error recovery, cooperative ARQ, data allocation

## Abstract

Energy harvesting wireless sensor networks (EH-WSNs) have been widely studied as a data collection infrastructure in the context of Artificial Intelligence of Things (AIoT). EH-WSNs face the challenge of achieving consistent data collection due to irregularly harvested environmental energy. Energy allocation and data allocation schemes have been proposed to balance energy consumption and data collection across the network; however, conventional error recovery techniques such as Automatic Repeat reQuest (ARQ) and Forward Error Correction (FEC) do not consider these allocation constraints, potentially leading to unintended energy depletion and data collection imbalance. In this paper, we propose a Cooperative ARQ (C-ARQ) scheme for EH-WSNs that incorporates energy allocation and data allocation. The proposed scheme computes the retransmittable data amount from the extra energy remaining after data allocation and performs retransmissions within that limit to recover errors, thereby preventing energy depletion and increasing the amount of data gathered at the sink node. Simulation results demonstrate that the proposed scheme improves the amount of data gathered at the sink node compared to other schemes, particularly in environments with longer hop paths, higher packet error rates, or more harvested energy.

## 1. Introduction

As Artificial Intelligence of Things (AIoT)—the convergence of Artificial Intelligence (AI) and the Internet of Things (IoT)—continues to advance [1,2], wireless sensor networks (WSNs) are widely used as a data collection infrastructure across diverse domains, including environmental monitoring, disaster detection, and smart agriculture [3,4]. Sensor nodes in WSNs are typically battery-powered and are often deployed in environments where battery replacement or recharging is difficult. As a result, minimizing energy consumption and extending network lifetime are among the key challenges in WSN research [5,6,7].

Energy harvesting technology has attracted attention as a means to overcome these energy constraints [8,9,10]. In energy harvesting-based WSNs (EH-WSNs), sensor nodes can operate continuously by harvesting energy from the environment. In particular, solar energy is one of the most widely used energy harvesting sources due to its high energy density [11,12]. However, since harvested solar energy varies significantly with time and season, energy prediction techniques [13,14,15] and energy allocation schemes can be employed to efficiently distribute the available energy [16,17,18]. Energy allocation limits the amount of energy usable per time slot, enabling relatively stable operation regardless of day or night. In addition to energy allocation, data allocation schemes can also be applied to address the hotspot problem [19,20,21], where data traffic concentrates near the sink node [22]. Data allocation distributes the transmission data amount fairly according to the available energy of each sensor node, preventing route disconnection due to energy depletion at specific nodes.

In WSNs, sensor nodes relay data to the sink node via multi-hop transmission, and the probability of transmission errors increases with increasing hop count. To address this, Automatic Repeat reQuest (ARQ), Forward Error Correction (FEC), and their combination, Hybrid ARQ (HARQ), have been studied [23]. However, traditional ARQ schemes cause frequent retransmissions when channel conditions deteriorate, resulting in significant energy consumption and transmission delays in resource-constrained sensor nodes. To overcome these limitations, Cooperative ARQ (C-ARQ) has emerged, exploiting the broadcast nature of wireless channels: neighboring nodes overhear packets destined for other nodes and retransmit them on behalf of the sender when errors occur [24]. C-ARQ not only provides spatial diversity through relay nodes to significantly improve link reliability, but also distributes the retransmission energy burden, which would otherwise be concentrated at the source node, among neighboring nodes. However, these error recovery schemes do not account for the additional energy overhead they impose and may consume more energy than ordinary data transmission. When applied together with energy allocation or data allocation schemes in WSNs, they may consume more energy than allocated or transmit less data than the allocated amount. This degrades the accuracy of energy and data allocation and reduces data collection efficiency and balance.

**Figure 1:**
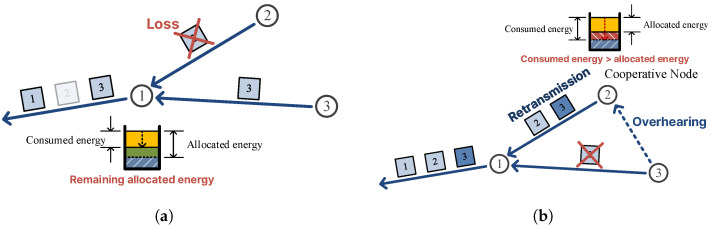
Motivating examples: (**a**) without error recovery, a transmission error at node 2 causes its parent node 1 to forward less data than planned, leaving allocated energy unused and reducing the amount of data gathered at the sink; (**b**) when a conventional C-ARQ scheme is applied without allocation awareness, cooperative node 2 retransmits on behalf of node 3, but consumes energy beyond its allocated budget, risking premature energy depletion and eventual route disconnection.

 Figure 1 illustrates the two problems that arise when error recovery is not properly integrated with data allocation. In  Figure 1a, a transmission error at node 2 prevents its data from reaching parent node 1, which consequently forwards less data than its planned quota to the sink; the allocated energy of node 1 is thus wasted and the overall data collection falls short. In  Figure 1b, cooperative node 2 successfully retransmits the lost data of node 3, recovering the error; however, node 2 expends more energy than its allocated budget in doing so, which can lead to energy depletion and route disconnection in subsequent rounds.

In this paper, we propose a novel scheme that integrates C-ARQ with energy allocation and data allocation in EH-WSNs. The proposed scheme recovers transmission errors within the allocated energy budget by utilizing the extra energy remaining after data allocation. Cooperative nodes are selected from among the neighbors of both the transmitting and receiving nodes; they overhear and temporarily store messages and then participate in retransmission within the retransmittable data limit, thereby preventing energy depletion while improving the amount of data gathered at the sink node.

The contributions of this paper are summarized as follows:

- We propose a method to utilize the extra energy arising from energy allocation and data allocation schemes for C-ARQ retransmission.
- We derive the retransmittable data amount from an energy model and design a cooperative retransmission scheme that operates within the allocated energy budget.
- We verify through simulation that the proposed scheme improves the amount of data gathered at the sink node compared to existing schemes.

The remainder of this paper is organized as follows. Section 2 reviews related work, and Section 3 describes the system model and detailed operation of the proposed scheme. Section 4 presents the performance evaluation results through simulation, and Section 5 concludes this paper.

## 2. Related Work

Energy harvesting is a key technology for addressing the lifetime problem of WSNs, and many researchers are actively investigating ways to reduce error rates by utilizing the energy harvested from the environment [23].

Jung et al. [25], Kang et al. [26], and Gil et al. [27] proposed energy-adaptive Reed–Solomon schemes using FEC to reduce the error rate in EH-WSNs. These schemes monitor the energy state of sensor nodes and dynamically adjust the parity length—using longer parity when the residual battery energy is sufficient and shorter parity when it is scarce—thereby reducing the data loss rate and optimizing network performance. However, these methods suffer from fixed energy thresholds, making it difficult to respond dynamically to changes in the network.

More recently, deep reinforcement learning (DRL) has been applied to optimize data collection parameters, such as charging duration and transmission rate, in real time for wirelessly powered IoT sensors [28]. However, this approach focuses on single-sensor optimal design and does not address the error recovery problem in multi-hop EH-WSNs operating under allocation constraints.

Jalali et al. [29] introduced the C-ARQ scheme to improve the reliability and energy efficiency of EH-WSNs. In this approach, a cooperative node located between the source and destination retransmits data on behalf of the source node, allowing the source to conserve energy for other tasks. However, since the cooperative node may be one of the candidate parent nodes of the source node, it risks consuming the parent node’s energy, which can lead to a degradation in the data delivery ratio.

Relay-assisted communications have also been studied in other domains; for instance, relay nodes have been utilized to improve the quality of demand response communications in smart grid networks, with cost modeling and game-theoretic strategies employed to optimize relay power allocation [30]. While such approaches demonstrate the versatility of relay-assisted designs, they target a fundamentally different network model and do not address the energy allocation constraints inherent in EH-WSNs.

Yang et al. [31] proposed SolarCode, a scheme that improves the reliability of data transmission in solar-powered WSNs by utilizing extra energy that is inevitably wasted when the battery is fully charged. This scheme dynamically adjusts the redundancy level of erasure coding based on per-slot solar energy harvesting predictions and battery state, thereby formulating an optimization problem that simultaneously guarantees a non-blackout network lifetime and maximizes the end-to-end packet delivery probability. However, when energy is insufficient on a specific link, the redundancy adjustment becomes limited, and the scheme is not suitable for scenarios where the amount of data collected is dynamically regulated.

Wu et al. [32] presented an adaptive redundancy-based mechanism to improve the reliability of data collection and minimize latency in WSNs. This scheme dynamically adjusts the redundant transmission of data packets based on the reliability and latency requirements of each link, demonstrating that reliable data delivery and optimized latency can be achieved. However, the scheme has a limitation in that the additional transmission overhead from redundant transmissions increases energy consumption, which can negatively affect energy-constrained nodes.

Bhanipati et al. [33] proposed Probabilistic Retransmission (PRT) and collision-impact-aware Probabilistic Retransmission (PRT-CI) schemes to minimize data collisions and improve energy efficiency in WSNs. These schemes dynamically adjust the retransmission of data packets based on each node’s data reception probability and collision likelihood, demonstrating that reliable data collection and optimized energy consumption can be achieved. However, the work focuses on collision phenomena during data transmission and does not consider data collection balance or dynamic changes in the amount of data collected.

In a broader context, joint resource allocation under multiple constraints has been studied in federated edge learning networks, where storage capacity, channel quality, and training latency are jointly optimized through user scheduling and bandwidth allocation [34]. These efforts highlight that resource-aware joint allocation is a critical design consideration across diverse wireless network paradigms, motivating similar constraint-aware approaches in EH-WSNs.

Koch et al. [35] proposed a timer-based scheme for distributed selection of the optimal relay node responsible for retransmission when applying C-ARQ to EH-WSNs. A node that overhears a packet sets a timer based on its current battery level and maximum battery capacity. Nodes with more residual energy are assigned shorter timers, and the node whose timer expires first retransmits the packet, ensuring that nodes with more energy handle retransmissions. In this way, energy-rich nodes are selected as cooperative nodes, thereby increasing the network’s energy efficiency. However, while this approach can conserve energy in the short term, it may cause relay nodes to consume more energy in the long term and can lead to data collection imbalance. Furthermore, this scheme assumes a single-hop topology in which all sensor nodes are directly connected to the sink node, making it inapplicable to the multi-hop communication environment targeted in this paper. Moreover, since this scheme does not incorporate data allocation, a direct performance comparison with the proposed scheme is not feasible.

Existing studies have primarily focused on improving the reliability of individual links or controlling retransmissions based on simple residual energy levels and thus have not been organically integrated with data allocation schemes that collect data fairly across the entire network. In energy-constrained harvesting environments, cooperative retransmissions that exceed the planned data transmission amount can cause energy depletion at relay nodes, leading to severe data collection imbalance and route disconnections within the network. In contrast, the scheme proposed in this paper preemptively applies energy and data allocation and then utilizes the extra energy for cooperative retransmission while ensuring that the amount of transmitted data does not exceed the allocated data quota. This enables effective recovery from transmission errors without depleting relay node energy and ultimately allows for stable and balanced data collection throughout the network while adhering to the pre-planned data allocation.

## 3. Error Recovery Using C-ARQ

**Figure 2:**
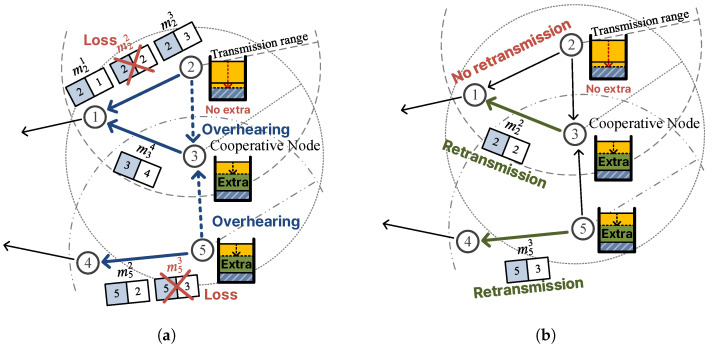
Overview of the proposed scheme: (**a**) during data transmission, a cooperative node overhears packets transmitted by neighboring nodes, and (**b**) a cooperative node with sufficient extra energy retransmits on behalf of a node that encounters a transmission error.

We propose a C-ARQ scheme for wireless sensor nodes with energy allocation and data allocation to reduce transmission errors. In this scheme, when a transmission error occurs, not only the sensor node that failed to transmit but also other sensor nodes that received the data cooperatively retransmit it, thereby increasing the data transmission success rate. In doing so, sensor nodes retransmit only within their allocated energy budget, preventing energy depletion and the consequent disruption of data transmission routes.  Figure 2 provides an overview of the proposed scheme.

### 3.1. Overall System Operation

The overall operation of the system proceeds as follows: (1) Sensor nodes perform routing every round of the period, $p_{\mathrm{round}}$, to determine the transmission route to the sink node. The proposed scheme uses the Minimum Depth Tree (MDT) routing algorithm [36] to minimize the number of hops to the sink node and thereby reduce transmission energy. (2) After the route is determined, all sensor nodes use the energy allocation scheme [16] and the data allocation scheme [22] to estimate the amount of data to be collected and determine the sensing interval accordingly. Each sensor node senses its surrounding environment at every interval to collect data and delivers it to the sink node via multi-hop transmission. (3) Relay nodes store the data they receive, forward it to their parent node, and broadcast an acknowledgment (ACK) message for the received data. Meanwhile, (4) if a node does not receive an ACK message for data it transmitted, it considers the data lost and retransmits the message; neighboring nodes that have previously received the message also retransmit it if they have sufficient extra energy. The following subsections describe each of these operations in detail.

### 3.2. Energy Model and Data Allocation

Since sensor nodes harvest solar energy as their energy source, the amount of energy collected varies with the time of day and season. In particular, no solar energy is harvested at night, so the energy collected during the day must be used throughout the night. If too much energy is consumed during the day, there may be insufficient energy for nighttime operation; if too little is consumed, the harvested energy may exceed the battery capacity and be lost. Therefore, a scheme that predicts the amount of harvested energy and uses it to determine energy consumption should be applied. In this paper, we adopt the Pro-Energy scheme [14] for energy prediction and the scheme proposed by Noh et al. [16] for energy allocation.

When sensor nodes forward sensed data to the sink node, a hotspot problem arises in which data concentrates at nodes near the sink. This causes severe energy depletion at nodes near the sink, making them prone to energy exhaustion and blackout, which can in turn sever the data transmission routes of other nodes and prevent data from reaching the sink. To address this issue, we apply our previous work, the data allocation scheme [22].

The data allocation scheme operates as follows: (1) Each node computes the maximum amount of data it can transmit based on its available energy and reports this to its parent node. (2) The parent node aggregates the transmittable amounts from all child nodes and allocates a fair data transmission quota to each child node. (3) Each child node that receives a quota uses it to compute and forward the data quota to its own child nodes, and (4) this process is repeated down to the leaf nodes to determine the data collection amount for the entire network. Through this process, data can be collected fairly from all nodes within the given energy budget.

In the data allocation scheme [22], if all nodes collect and transmit an equal amount of data $s_{\mathrm{sense}}$, the total amount of data ${s_{\mathrm{aggr}}}_{i}$ that node *i* aggregates from itself and its child nodes and transmits during one round is expressed as follows:

(1) $${s_{\mathrm{aggr}}}_{i}=|T_{i}|s_{\mathrm{sense}},$$

where $|T_{i}|$ is the number of nodes in the subtree rooted at node *i*, and $s_{\mathrm{sense}}$ is the amount of data sensed by a single node. If the energy allocated for one round is $e_{\mathrm{avail}}$, the energy consumed $e_{c}$ during this period should satisfy $e_{c}\leq e_{\mathrm{avail}}$, where $e_{c}$ includes the energy for data transmission, reception, and other operations. That is, for a sensor to operate within the allocated energy budget $e_{\mathrm{avail}}$, the following should hold:

(2) $$e_{\mathrm{avail}}\geq e_{\mathrm{Tx}}+e_{\mathrm{Rx}}+e_{\mathrm{idle}},$$

where $e_{\mathrm{Tx}}$ is the energy for transmitting data, $e_{\mathrm{Rx}}$ is the energy for receiving data (including overhearing energy), and $e_{\mathrm{idle}}$ is the energy consumed for all purposes other than $e_{\mathrm{Tx}}$ and $e_{\mathrm{Rx}}$. $e_{\mathrm{Tx}}$ is determined by the amount of sensed data transmitted and the amount of ACKs transmitted. The sensed data transmission amount is $s_{\mathrm{sense}}|T_{i}|$, since each node must forward $s_{\mathrm{sense}}$ for itself and all its descendant nodes. For ACK transmission, if ACKs for all received data are batched and sent at once with up to $R_{\max}$ retransmissions, at most $R_{\max}\left(|T_{i}|-1\right)$ ACKs of size $s_{\mathrm{ack}}$ are transmitted. Using the energy model of Melodia et al. [37], $e_{\mathrm{Tx}}$ is expressed as follows:

(3) $$e_{\mathrm{Tx}}=\left(s_{\mathrm{sense}}|T_{i}|+R_{\max}s_{\mathrm{ack}}\left(|T_{i}|-1\right)\right)\beta d^{\alpha },$$

where α is the path loss exponent, which typically ranges from 2 to 5 depending on the deployment environment (e.g., 2.7–3.0 for general outdoor, 3.0–3.5 for indoor office, and 4.0 or above for industrial environments [38,39], β is the energy consumption per bit per unit distance ($J/\mathrm{bit}/m^{\alpha }$), and d is the transmission distance (m). By substituting $e_{\mathrm{Tx}}$ from Equation (3) into Equation (2) and solving for $s_{\mathrm{sense}}$, $s_{\mathrm{sense}}$ must satisfy the following:

(4) $$s_{\mathrm{sense}}\leq \frac{e_{\mathrm{avail}}-e_{\mathrm{Rx}}-e_{\mathrm{idle}}}{|T_{i}|\beta d^{\alpha }}-\frac{R_{\max}s_{\mathrm{ack}}\left(|T_{i}|-1\right)}{|T_{i}|}.$$

This $s_{\mathrm{sense}}$ is substituted into Equation (1) to compute the total data amount $s_{\mathrm{aggr}}$ that the node should transmit, which is then reported to the parent node so that the parent can compute its own ${s_{\mathrm{aggr}}}_{i}$.

In this data allocation scheme, the ${s_{\mathrm{aggr}}}_{i}$ computed at the parent node is used to derive the fair per-node transmittable data amount $s_{\mathrm{sense}}$, which is then delivered to child nodes so that an equal amount of data is collected. That is, from Equation (1), the amount of data ${s_{\mathrm{sense}}}_{i}$ that can be sensed by parent node *i* and its child nodes is expressed as follows:

(5) $${s_{\mathrm{sense}}}_{i}=\frac{{s_{\mathrm{aggr}}}_{i}}{|T_{i}|}.$$

The computed ${s_{\mathrm{sense}}}_{i}$ is delivered to each child node, which uses it to update its own ${s_{\mathrm{aggr}}}_{j}$ and compute ${s_{\mathrm{sense}}}_{j}$ to pass further down to its child nodes. By repeating this process, the data collection amount for every node in the network can be determined.

Once ${s_{\mathrm{sense}}}_{j}$ is determined, each sensor node uses it to set the sensing interval $p_{\mathrm{sense}}$ for one round. When the length of one round is $p_{\mathrm{round}}$, the sensing interval ${p_{\mathrm{sense}}}_{j}$ of node *j* is represented as follows:

(6) $${p_{\mathrm{sense}}}_{j}=p_{\mathrm{round}}\frac{s_{\mathrm{unit}}}{{s_{\mathrm{sense}}}_{j}},$$

where $s_{\mathrm{unit}}$ denotes the size of data sensed in a single measurement. In this way, every node senses and transmits data at its own sensing interval $p_{\mathrm{sense}}$, enabling relatively fair data collection within a limited energy budget.

**Figure 3:**
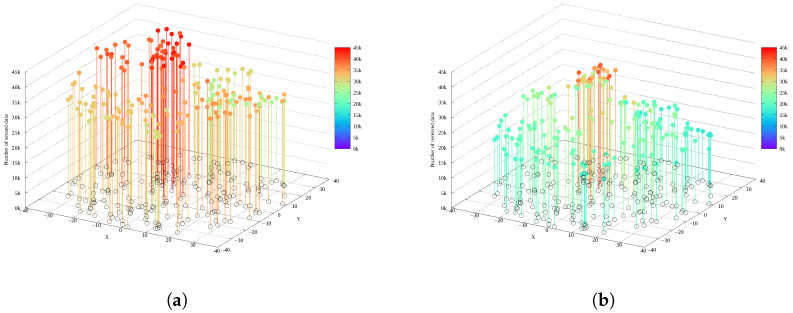
Distribution of sensed data amount and data received at the sink node: (**a**) distribution of sensed data amount and (**b**) distribution of data amount received at the sink node.

When the data allocation scheme is applied, nodes farther from the sink tend to have relatively more remaining energy. Furthermore, due to the nature of multi-hop transmission, the error rate increases as data traverses more hops, so the amount of data collected from nodes far from the sink decreases.  Figure 3 shows the distribution of data collected in this manner. To exploit this, we aim to utilize the extra energy $e_{\mathrm{extra}}$ for retransmission to improve the transmission success rate. To this end, $e_{\mathrm{extra}}$ must be derived.

By considering the energy consumed $e_{\mathrm{used}}$ and the allocated energy $e_{\mathrm{avail}}$ during one round, $e_{\mathrm{extra}}$ is calculated as follows:

(7) $$e_{\mathrm{extra}}=e_{\mathrm{avail}}-e_{\mathrm{used}},$$

where $e_{\mathrm{used}}$ includes not only the energy for data transmission, reception, and idle state, but also all other energy consumption such as ACK messages and overhearing. It can be obtained by substituting ${s_{\mathrm{sense}}}_{i}$ determined from Equation (5) into Equations (2) and (3). Substituting this into the energy model of Melodia et al. [37] via Equation (7), the retransmittable data amount $s_{\mathrm{ret}}$ is represented as follows:

(8) $$s_{\mathrm{ret}}=\frac{e_{\mathrm{extra}}}{\beta d^{\alpha }}.$$

**Figure 4:**
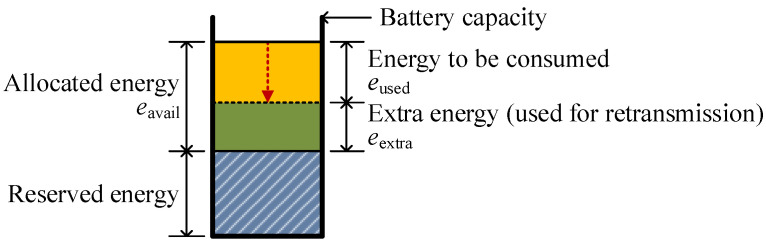
Energy model of the proposed scheme.

That is, a sensor node can retransmit up to $s_{\mathrm{ret}}$ amount of data during one round.  Figure 4 illustrates the proposed energy model.

### 3.3. Retransmission Operation

In this paper, we propose an improved C-ARQ scheme tailored to the data allocation framework. Similarly to a standard ARQ scheme, a sensor node that receives a message transmits an ACK in response. To reduce the number of packets and transmission energy, ACKs for messages received over a given period are batched and sent at once. Specifically, each node inserts the acknowledgment information $r_{j}^{l}$ for each message received during that period into an ACK message set A. $r_{j}^{l}$ is a tuple (j,l), where *j* is the ID of the node that transmitted the sensed data and *l* is the sequence number of the message. The node broadcasts A, and a node that previously transmitted a message determines that its transmission was successful if it finds the corresponding $r_{j}^{l}$ in the received A. If a node fails to find an ACK for its message in A, or does not receive A at all, it retransmits the message. To ensure energy consumption stays within the allocated budget, a sensor node checks $s_{\mathrm{ret}}$ before retransmitting and retransmits only if sufficient extra energy is available.

The cooperative retransmission operates in an event-driven manner, responding to three events: packet overhearing, ACK reception, and retransmission timeout. The key design principles are: (i) a cooperative node selectively stores only packets whose sender and receiver are both reachable neighbors, ensuring retransmission feasibility; (ii) retransmission is performed only up to $s_{\mathrm{ret}}$, ensuring that the allocated energy budget is not exceeded; and (iii) an $e_{\mathrm{extra}}$-based backoff is applied at retransmission time to avoid packet collisions and excessive duplicate retransmissions. In addition to the ARQ described above, a cooperative sensor node performs retransmission on behalf of the original sender. A cooperative sensor node is one that is a neighbor of both the sender and the receiver of the message and has sufficient $s_{\mathrm{ret}}$. The cooperative sensor node *i* performs C-ARQ as follows. (1) Sensor node *i* builds a neighbor node set $N_{i}$ during the routing process. By reusing the routing messages already exchanged during route setup, no additional control overhead is required to discover neighbors. (2) When a neighboring sensor node *j* broadcasts data $m_{j}^{l}$ to sensor node *k* and node *i* overhears it, node *i* checks whether both the sender *j* and the destination *k* belong to $N_{i}$. This condition ensures that node *i* has direct communication links to both parties, so that a retransmission from node *i* can actually reach the intended destination. (3) If both *j* and *k* are in $N_{i}$, node *i* temporarily stores $m_{j}^{l}$ and adds $r_{j}^{l}$ to the pending retransmission set $M_{i}$. Although the number of messages to be stored can increase with the number of sender nodes *j* and their descendant nodes, the storage is limited to messages whose sender *j* and destination *k* are both in $N_{i}$. Furthermore, since each sensed data message is only a few to tens of bytes, these two factors together ensure that the memory overhead remains negligible even in dense networks. (4) When node *k* successfully receives $m_{j}^{l}$, it adds the corresponding ACK $r_{j}^{l}$ to $A_{k}$ and broadcasts $A_{k}$ after a certain period. (5) When node *i* receives $A_{k}$, it removes the acknowledged entries from $M_{i}$, canceling any pending retransmission for already-delivered packets and thereby avoiding unnecessary energy consumption, as follows:

(9) $$M_{i}=M_{i}-A_{k}.$$

(6) After a timeout, if $r_{j}^{l}$ still remains in $M_{i}$, node *i* acts as a cooperative node and retransmits the temporarily stored $m_{j}^{l}$. To prevent simultaneous transmissions from multiple cooperative nodes causing collisions, and to avoid duplicate retransmissions of the same message from different nodes, we adopt an energy-based backoff scheme inspired by LEACH [40]. A node with more retransmittable energy $e_{\mathrm{extra}}$ employs a shorter backoff time and thus transmits before other nodes, thereby avoiding duplicate transmissions and packet collisions. Algorithm 1 illustrates the above procedure.
**Algorithm 1** Cooperative ARQ at cooperative node *i*  1:**Initialization:**  2:    $N_{i}\leftarrow$ neighbor nodes obtained from routing  3:    $M_{i}\leftarrow \emptyset$  4:**procedure** OnOverhear($m_{j}^{l}$, sender *j*, destination *k*)  5:      **if** $j\in N_{i}$ **and** $k\in N_{i}$ **then**             ▹ Feasibility check  6:            store $m_{j}^{l}$ temporarily  7:            $M_{i}\leftarrow M_{i}\cup \left\{r_{j}^{l}\right\}$         ▹ Track pending retransmission  8:      **end if**  9:**end procedure**10:**procedure** OnACKReceived($A_{k}$ from node *k*)11:      $M_{i}\leftarrow M_{i}-A_{k}$               ▹ Cancel delivered packets12:**end procedure**13:**procedure** OnTimeout14:      **for all** $r_{j}^{l}\in M_{i}$ **do**15:            **if** $s_{\mathrm{ret}}\geq \left|m_{j}^{l}\right|$ **then**             ▹ Energy budget check16:                  wait for energy-proportional backoff17:                  **if** $r_{j}^{l}$ still in $M_{i}$ **then**18:                        retransmit stored $m_{j}^{l}$ to node *k*19:                  **end if**20:            **end if**21:      **end for**22:**end procedure**

**Figure 5:**
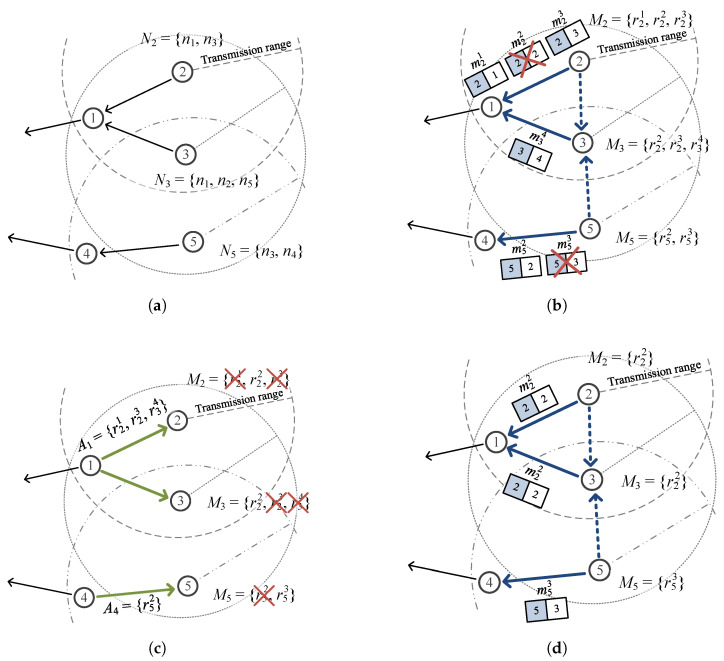
Example of the retransmission process: (**a**) generation of the neighbor node set *N* during routing, (**b**) transmission of sensing data and temporary storage by the receiving node, (**c**) removal of acknowledged entries from *M* upon ACK reception, and (**d**) retransmission of data corresponding to remaining entries in *M*.

We illustrate the proposed scheme with an example using Figure 5. (a) First, during the routing process, each node exchanges routing messages with neighboring nodes and adds those nodes to its neighbor set *N*. The figure shows node $n_{3}$ exchanging routing messages with $n_{1}$, $n_{2}$, and $n_{5}$ to form $N_{3}=\left\{n_{1},\;n_{2},\;n_{5}\right\}$. (b) Each sensor node delivers its sensed data to the sink node via multi-hop transmission. The figure shows $n_{2}$ transmitting $m_{2}^{1}$, $m_{2}^{2}$, and $m_{2}^{3}$ and $n_{3}$ transmitting $m_{3}^{4}$ to $n_{1}$, and $n_{5}$ transmitting $m_{5}^{2}$ and $m_{5}^{3}$ to $n_{4}$. Here, $m_{2}^{2}$ and $m_{5}^{3}$ fail to be delivered. Since $n_{3}$ can also overhear the data transmitted by $n_{2}$ and $n_{5}$, it checks whether the sender and receiver of each overheard message are in its neighbor set and, if so, temporarily stores the message and adds the corresponding entry to $M_{3}$. Since both $n_{1}$ and $n_{2}$ are neighbors of $n_{3}$, node $n_{3}$ temporarily stores the messages from $n_{2}$ and adds $r_{2}^{2}$ and $r_{2}^{3}$ to $M_{3}$. Note that $r_{2}^{1}$ is not added because $m_{2}^{1}$ is assumed to have failed to reach $n_{3}$. Meanwhile, although $n_{5}$ is a neighbor of $n_{3}$, $n_{4}$ is not, so $n_{3}$ neither stores the messages from $n_{5}$ nor adds anything to $M_{3}$. (c) Nodes that successfully receive messages broadcast their ACK messages. In the figure, $n_{1}$ broadcasts $A_{1}=\left\{r_{2}^{1},r_{2}^{3},r_{3}^{4}\right\}$ for $m_{2}^{1}$, $m_{2}^{3}$, and $m_{3}^{4}$, and $n_{4}$ broadcasts $A_{4}=\left\{r_{5}^{2}\right\}$ for $m_{5}^{2}$. Nodes that receive these ACKs perform M=M−A; thus, $n_{3}$ receives $A_{1}$ and its set becomes $M_{3}=\left\{r_{2}^{2}\right\}$. (d) If a sensor node has remaining $s_{\mathrm{ret}}$, it retransmits the messages corresponding to entries still in M. In the figure, $n_{2}$ retransmits $m_{2}^{2}$ and $n_{5}$ retransmits $m_{5}^{3}$. As a cooperative node, $n_{3}$ also retransmits the temporarily stored $m_{2}^{2}$, thereby increasing the transmission success rate.

By performing retransmission through this process, each node can retransmit not only its own data but also the data of neighboring nodes within its retransmission budget, without depleting its own energy. At the same time, it transmits the planned data amount without exceeding the transmission limit of the parent node, while increasing the overall transmission success rate.

## 4. Performance Evaluation

To evaluate the performance of the proposed scheme, we conducted simulations under various network conditions. The simulations were performed using SolarCastalia [41]. All simulation logic and configurations were implemented in C++. Each simulation scenario was independently repeated 100 times with different random seeds, and all results are presented as averages over these runs. Error bars in all figures represent 95% confidence intervals.

We compare the proposed scheme against the following five EH-WSN schemes:

- Naive (No Retransmission): A standard WSN scheme without energy/data allocation or error recovery. This represents the most common WSN deployment and allows us to evaluate the overall benefit of the proposed scheme over a general-purpose WSN.
- Naive (ARQ): A standard WSN scheme without energy/data allocation but with conventional ARQ for error recovery. This scheme isolates the effect of error recovery alone and allows us to assess how much additional gain the proposed scheme achieves over a simple error recovery approach.
- Data Allocation (No Error): A scheme with data allocation applied under an ideal zero-error channel assumption. This represents the theoretical upper bound of data collection achievable with data allocation, against which the proposed scheme can be compared to assess how closely it approaches the ideal.
- Data Allocation (No Retransmission): A scheme with data allocation but no error recovery. This scheme demonstrates the degradation caused by transmission errors in a data allocation-enabled network and quantifies the performance gain attributable solely to the error recovery mechanism of the proposed scheme.
- Data Allocation (ARQ): A scheme combining data allocation with conventional ARQ, where retransmissions are performed without regard to allocation constraints. This scheme shows the performance of a naive combination of data allocation and ARQ, highlighting the advantage of the proposed allocation-aware C-ARQ over a straightforward ARQ approach.

For performance evaluation, we measure the cumulative amount of data sensed by each sensor node and how much of that sensed data reaches the sink node. The simulated WSN consists of 1000 energy harvesting sensor nodes and a single sink node, randomly deployed in the field. Table 1 lists the key simulation parameters.

### 4.1. Performance Comparison by Total Number of Nodes

**Figure 6:**
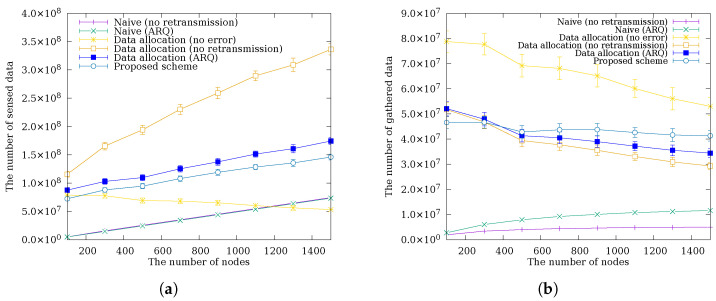
The amount of (**a**) sensed data and (**b**) data gathered at the sink node according to the total number of nodes.

Figure 6 shows a comparison of the amount of sensed data and data gathered at the sink node as the total number of nodes varies. In Figure 6a, the total amount of sensed data generally increases with the number of nodes across all schemes. A notable exception is the Data Allocation (No Error) scheme, which shows a decrease in sensed data as the number of nodes grows. This occurs because a larger node count increases the hop depth of the tree, causing relay nodes near the sink to aggregate and forward data from a greater number of descendant nodes; the resulting energy burden on near-sink nodes tightens the data allocation budget for the entire subtree, ultimately reducing per-node sensing quotas. The other data allocation schemes show a more moderate increase in sensed data with node count. Because transmission errors cause some forwarded packets to be lost, relay nodes expend less energy than in the error-free case, leaving a larger residual budget that the data allocation scheme redistributes as additional sensing quota. In Figure 6b, the Naive and Naive (ARQ) schemes gather significantly less data regardless of the number of nodes. Without data allocation, the hotspot problem concentrates relay traffic at near-sink nodes regardless of total node count, leading to energy depletion at those nodes and persistent route disruption; increasing the number of nodes therefore does not improve the amount of data gathered at the sink. In contrast, schemes with data allocation maintain relatively stable data gathering regardless of the number of nodes by distributing the energy burden evenly. Among these, the proposed scheme shows slightly lower data gathering than other data allocation schemes when the number of nodes is small because shorter hop paths reduce the compounding of per-hop errors, making the benefit of cooperative retransmission marginal while its energy overhead remains. As the number of nodes increases, however, packet errors accumulate multiplicatively along longer hop paths, and the cooperative retransmission of the proposed scheme recovers an increasing share of these errors, yielding a growing performance advantage over other data allocation schemes. Unlike Data Allocation (ARQ), where only the original sender retransmits, C-ARQ provides an additional recovery path through a cooperative neighbor: when the original sender lacks sufficient $e_{\mathrm{extra}}$ to retransmit, a cooperative node with available $e_{\mathrm{extra}}$ can retransmit on its behalf, increasing the probability of successful delivery along longer hop paths.

### 4.2. Performance Comparison by Node Density

**Figure 7:**
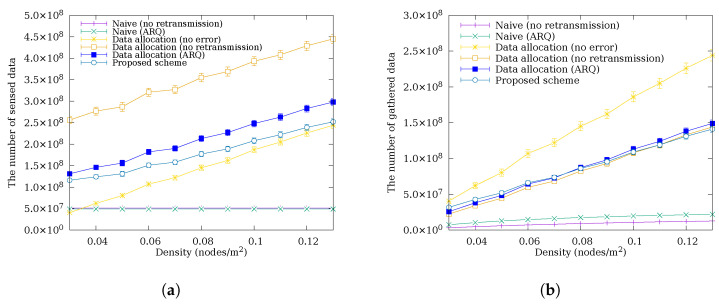
The amount of (**a**) sensed data and (**b**) data gathered at the sink node according to the node density.

Figure 7 shows a comparison of the amount of sensed data and data gathered at the sink node as the node density varies. In Figure 7a, unlike schemes without data allocation, schemes with data allocation show an increase in sensed data as density increases. The results indicate that higher density reduces the number of hops, which in turn reduces the impact of packet errors and the energy consumption of relay nodes, allowing more data to be allocated. In Figure 7b, the proposed scheme shows good performance for densities from 0.03 to 0.06 but shows slightly lower performance than Data Allocation (ARQ) from 0.07 onward. At high densities, the hop count drops to one or two, reducing the end-to-end error rate to near single-hop PER levels. Under these conditions, the benefit of cooperative retransmission becomes negligible, while the energy overhead of overhearing and backoff introduces a net loss, causing the proposed scheme to fall slightly below Data Allocation (ARQ). At lower densities, longer hop paths cause packet errors to accumulate. The additional recovery path provided by C-ARQ through cooperative neighbors, each retransmitting within their own $e_{\mathrm{extra}}$, increases the probability of successful delivery beyond what single-sender Data Allocation (ARQ) can achieve, and the proposed scheme outperforms all other schemes except the error-free case.

### 4.3. Performance Comparison by Packet Error Rate

**Figure 8:**
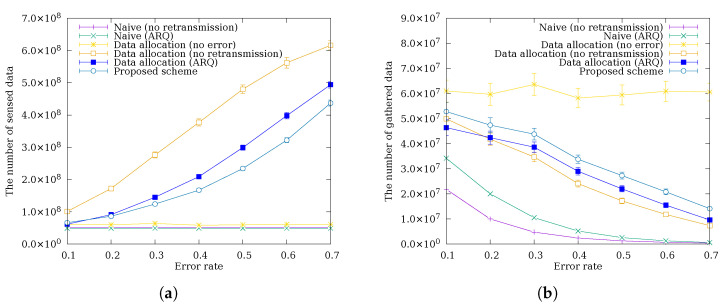
The amount of (**a**) sensed data and (**b**) data gathered at the sink node according to the packet error rate.

Figure 8 shows a comparison of the amount of sensed data and data gathered at the sink node as the packet error rate varies. This simulation assumes a uniform packet error rate across all links. This model has the limitation of not reflecting the varying error rates due to distance, fading, and interference, which affect link quality in practical deployments. Nevertheless, experiments across a range of packet error rate conditions partially compensate for this limitation. In Figure 8a, schemes without data allocation sense a constant amount of data regardless of the packet error rate because they use a fixed sensing interval that does not adapt to channel conditions. In contrast, schemes with data allocation show an increase in sensed data as the packet error rate increases. A higher error rate causes more packets to be lost in transit, reducing the amount of data that relay nodes actually forward and thereby lowering their energy consumption. The data allocation scheme detects this residual energy and redistributes it as additional sensing quota, resulting in more sensed data at elevated error rates. Notably, the proposed scheme senses less data than Data Allocation (ARQ) in (a). This is because the superior error recovery of the proposed scheme causes a higher fraction of sensed data to successfully reach the sink, leading relay nodes to forward more data and consume more energy; the data allocation scheme consequently assigns a lower sensing quota to stay within the energy budget. In Figure 8b, all schemes except the error-free one show a decrease in gathered data as the packet error rate increases. Schemes without error recovery exhibit a much steeper decline because each lost packet is permanently unrecoverable and losses accumulate directly into reduced data gathering. Despite sensing less data in (a), the proposed scheme gathers more data at the sink than Data Allocation (ARQ) because C-ARQ provides an additional recovery path through a cooperative neighbor: when the original sender lacks sufficient $e_{\mathrm{extra}}$ to retransmit, a cooperative node with available $e_{\mathrm{extra}}$ retransmits on its behalf, achieving a higher delivery ratio than the single-sender retransmission of Data Allocation (ARQ). Furthermore, as the packet error rate increases, the absolute amount of data recovered through this additional path grows, widening the performance gap between the proposed scheme and other schemes.

### 4.4. Performance Comparison by Harvested Energy

**Figure 9:**
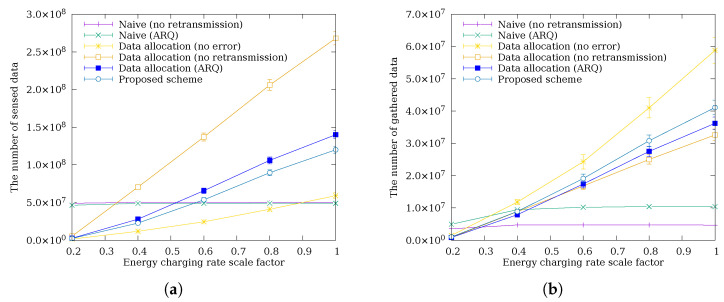
The amount of (**a**) sensed data and (**b**) data gathered at the sink node according to the harvested energy.

Figure 9 shows a comparison of the amount of sensed data and data gathered at the sink node as the amount of harvested energy varies. When the harvested energy is low, schemes without data allocation gather more data. Under energy-scarce conditions, data allocation simultaneously constrains all nodes to very low sensing quotas, suppressing overall data gathering. In contrast, schemes without data allocation allow some nodes to go idle and accumulate energy, which other nodes then use to gather bursts of additional data; the total amount is higher, but the gathering is temporally and spatially imbalanced. As the harvested energy increases, schemes with data allocation gather more data by redistributing the growing energy budget evenly across nodes. In particular, the proposed scheme senses less data than other data allocation schemes but gathers more at the sink. As harvested energy increases, the extra energy budget $e_{\mathrm{extra}}$ available for retransmission also grows, enabling C-ARQ to recover a greater number of failed packets. This amplifies the error recovery benefit with increasing energy, causing the advantage of the proposed scheme over other data allocation schemes to grow. While Data Allocation (ARQ) retransmits unconditionally without checking the remaining energy budget, which can cause relay nodes to exceed their allocated budget and risk energy depletion, the proposed scheme restricts retransmission to within $e_{\mathrm{extra}}$, preventing such depletion and maintaining stable routes. Furthermore, cooperative nodes contribute their own $e_{\mathrm{extra}}$ for retransmission, providing a larger collective recovery capacity. As harvested energy increases and $e_{\mathrm{extra}}$ grows, this budget-aware cooperative retransmission recovers more failed packets while sustaining stable operation, increasingly outperforming the unconditional ARQ approach.

### 4.5. Fairness and Energy Efficiency Analysis

**Figure 10:**
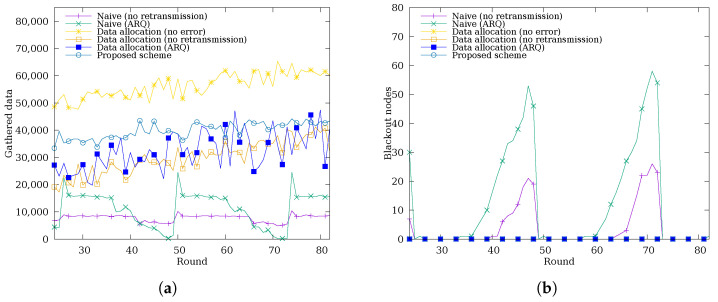
Time-series of (**a**) data gathered at the sink node per round and (**b**) number of blackout nodes per round over the 3-day simulation.

Figure 10 shows the amount of data gathered at the sink node per round and the number of blackout nodes per round over the 3-day simulation to evaluate how fairly data is gathered over time. As shown in Figure 10b, the naive schemes without data allocation exhibit periodic occurrences of blackout nodes. This is attributed to the depletion of energy in some nodes during nighttime, when no solar energy is harvested. Accordingly, as shown in Figure 10a, the naive schemes gather less data overall; in particular, Naive (ARQ) shows large fluctuations in the amount of data gathered over time. In contrast, schemes with data allocation produce no blackout nodes even during nighttime, resulting in relatively stable data gathering regardless of the time of day. In particular, while the proposed scheme does not reach the performance of the ideal case (Data Allocation (No Error)), it gathers more data more stably than the other schemes.

**Figure 11:**
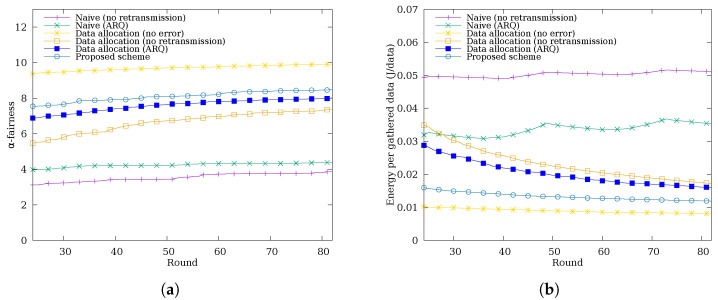
Time-series of (**a**) α-fairness of cumulative data gathered at the sink node per node and (**b**) energy consumption per successfully gathered data packet over simulation rounds.

To evaluate the spatial fairness of the proposed scheme, we measure the α-fairness [42] of the cumulative data successfully delivered to the sink node per sensor node, using α=1 to balance fairness and efficiency. Unlike a direct comparison of total gathered data, α-fairness quantifies fairness by weighting each node’s contribution logarithmically, penalizing large disparities in per-node delivery. As shown in Figure 11a, the naive schemes without data allocation exhibit lower α-fairness, as periodic blackout nodes result in highly uneven cumulative data delivery across nodes. In contrast, schemes with data allocation maintain higher fairness by preventing blackouts and distributing the data collection load more evenly across all nodes. The Data Allocation (No Error) scheme achieves the highest fairness, which is attributed to the absence of transmission errors, ensuring that all nodes successfully deliver their allocated data quotas, resulting in minimal inter-node disparity. The proposed scheme ranks second, achieving fairness comparable to the Data Allocation (ARQ) scheme, indicating that the primary driver of fairness improvement is the data allocation mechanism, with cooperative retransmission providing a modest additional gain.

Figure 11b shows the energy consumption per successfully gathered data packet over simulation rounds, where a lower value indicates higher energy efficiency. The Naive (No Retransmission) scheme exhibits the worst energy efficiency by a large margin, as transmission errors go unrecovered and the energy spent on unsuccessful transmissions is entirely wasted, as no recovery mechanism exists to compensate for lost packets. The Naive (ARQ) scheme shows the second-lowest efficiency; while retransmission recovers some errors, the lack of data allocation concentrates traffic load at nodes near the sink, resulting in poor energy utilization. Schemes with data allocation achieve comparatively higher efficiency, and among them, better error recovery capability leads to better energy efficiency. The Data Allocation (No Error) scheme achieves the best efficiency; since no transmission errors occur, all expended energy contributes directly to successful data delivery. The proposed scheme achieves higher energy efficiency than Data Allocation (ARQ), despite both using data allocation. Data Allocation (ARQ) retransmits unconditionally without checking the remaining energy budget, which can cause relay nodes to exceed their allocated budget and waste energy on budget overruns. In contrast, the proposed scheme retransmits only within $e_{\mathrm{extra}}$, avoiding such waste, while cooperative nodes additionally contribute their own $e_{\mathrm{extra}}$ to deliver more data per unit of energy consumed. This budget-aware cooperative retransmission results in energy efficiency close to the ideal case.

The evaluation results show that the proposed scheme outperforms other schemes in environments with increasing hop counts, low node density where packet errors accumulate easily, high packet error rates, and large amounts of harvested energy, as the benefit of budget-aware cooperative retransmission grows with the impact of errors. In environments with very high node density or few hops, the error impact is minimal and the energy overhead of retransmission becomes relatively prominent, causing the proposed scheme to perform similarly to or slightly below Data Allocation (ARQ). Beyond total data collection, the proposed scheme also achieves higher spatial fairness and better energy efficiency than schemes without data allocation. In particular, by restricting retransmission to within the allocated energy budget and leveraging cooperative nodes, the proposed scheme avoids the energy depletion caused by unconditional retransmission in Data Allocation (ARQ), resulting in energy efficiency close to the ideal error-free case. These results demonstrate the practicality of the proposed scheme across diverse EH-WSN deployment scenarios.

## 5. Conclusions

In this paper, we proposed a novel scheme that integrates C-ARQ with data allocation in EH-WSNs. Without proper integration, two problems arise: without error recovery, transmission errors waste the allocated energy of relay nodes, and conventional C-ARQ without allocation awareness causes cooperative nodes to exceed their energy budget, risking depletion. The proposed scheme derives a retransmittable data quota from the energy model and performs retransmission strictly within this budget. Cooperative nodes are selected from among the nodes that neighbor both the sender and receiver; they overhear and store packets from neighboring transmissions and retransmit upon timeout within their own retransmittable quota, collectively increasing recovery capacity without depletion risk. Simulation results confirmed that the performance advantage over other schemes grows with longer hop paths, higher packet error rates, and more harvested energy, while the proposed scheme also achieves higher spatial fairness and better energy efficiency. As future work, we plan to extend the scheme to adaptively recover errors in short-hop or energy-scarce environments and to explore more sophisticated cooperative node selection and a hybrid approach combining C-ARQ with FEC.

## Figures and Tables

**Table 1:** Simulation parameters.

Parameters	Values
Number of nodes	1000
Node density	0.04
Topology	Random
Duration of a round	1 h
Battery capacity	47 mAh
Sensory data size	8 bytes
Packet error rate	0.3
Baud rate	250 kbps
Solar power density	$15\;{\mathrm{mW/cm}}^{2}$
α	3
β	$10^{-6}$ ${\mathrm{J/bit/m}}^{3}$
$p_{\mathrm{round}}$	1 h
Simulation time	7 days
Number of simulation runs	100
Confidence interval	95%

## Data Availability

The original contributions presented in this study are included in the article. Further inquiries can be directed to the author.

## Abbreviations

The following abbreviations are used in this manuscript:

ACK	Acknowledgement
AIoT	Artificial Intelligence of Things
ARQ	Automatic Repeat reQuest
C-ARQ	Cooperative ARQ
EH-WSN	Energy harvesting wireless sensor network
HARQ	Hybrid ARQ
MDT	Minimum Depth Tree
PRT	Probabilistic Retransmission
WSN	Wireless sensor network
